# Peroxiredoxins in Stroke: Friends and Foes

**DOI:** 10.3390/cells15070640

**Published:** 2026-04-02

**Authors:** Yingfeng Wan, Jingwei Zhang, Liheng Bian, Xiaoxiao Tan, Ting Chen, Guohua Xi, Ya Hua, Aditya S. Pandey, Richard F. Keep, Sravanthi Koduri

**Affiliations:** Department of Neurosurgery, University of Michigan, Ann Arbor, MI 48109, USA

**Keywords:** peroxiredoxin, hemorrhagic stroke, ischemic stroke, oxidative stress, neuroinflammation

## Abstract

Stroke, a leading cause of mortality and long-term disability, induces complex cascades of oxidative stress and neuroinflammation that exacerbate brain injury. The peroxiredoxin (Prx; Prdx) family, composed of six thiol-dependent antioxidant enzymes (Prx1–6), plays a pivotal role in regulating redox homeostasis and immune responses in the brain. This review synthesizes current knowledge on the isoform-specific roles of Prxs in both ischemic and hemorrhagic stroke, highlighting their dual and context-dependent functions. Intracellular Prxs generally protect neurons and maintain blood–brain barrier (BBB) integrity, while extracellular Prxs—particularly Prx1 and Prx2—act as damage-associated molecular patterns (DAMPs), activating toll-like receptor signaling and amplifying inflammation. Isoforms such as Prx3 and Prx4 exhibit mitochondrial and endothelial protective effects, respectively, whereas Prx6 demonstrates complex roles influenced by its acidic, Ca^2+^-independent, phospholipase A2 (aiPLA2) activity and cellular localization. We also discuss emerging tools for studying Prx biology and explore the translational potential of Prxs as biomarkers and therapeutic targets. Ultimately, a nuanced understanding of Prx dynamics offers new avenues for stroke diagnosis, prognosis, and treatment.

## 1. Introduction

Stroke is a leading cause of death and long-term disability worldwide and can be broadly classified into two major types: ischemic stroke and hemorrhagic stroke. Ischemic stroke, which accounts for approximately 85% of all cases, results from the obstruction of cerebral blood flow due to thrombotic or embolic events, leading to oxygen and nutrient deprivation in brain tissue [1]. In contrast, hemorrhagic stroke, which includes intracerebral hemorrhage (ICH), intraventricular hemorrhage (IVH), and subarachnoid hemorrhage (SAH), arises from the rupture of weakened blood vessels, causing bleeding into or around the brain parenchyma [2,3]. Both types of strokes initiate complex cascades of cellular injury, oxidative stress, inflammation, and blood–brain barrier (BBB) disruption, ultimately contributing to neuronal death and neurological dysfunction. Understanding the distinct and overlapping mechanisms underlying these stroke subtypes is critical for developing effective therapeutic strategies, including those targeting oxidative stress-related pathways.

Oxidative stress and neuroinflammation are central pathological processes in both ischemic and hemorrhagic stroke [4,5]. Following cerebral injury, excessive production of reactive oxygen species (ROS) overwhelms endogenous antioxidant defenses, leading to lipid peroxidation, deoxyribonucleic acid (DNA) damage, and protein oxidation, which contribute to neuronal death and BBB disruption [6]. Concurrently, activation of resident microglia and infiltration of peripheral immune cells amplify the inflammatory response, releasing proinflammatory cytokines, chemokines, and matrix metalloproteinases that exacerbate brain tissue damage [7]. This interplay between oxidative stress and neuroinflammation forms a self-perpetuating cycle that accelerates neuronal injury and impedes recovery. Understanding the molecular mediators that regulate redox balance and inflammatory signaling is essential for identifying therapeutic targets. Among these, the peroxiredoxin (Prx) family has emerged as a key player, linking antioxidant defense with immune modulation in the context of stroke.

Prxs are a family of highly conserved, thiol-dependent antioxidant enzymes that play crucial roles in maintaining cellular redox homeostasis and protecting against oxidative stress [8,9]. In mammals, six isoforms (Prx1–Prx6) have been identified, each with distinct subcellular localizations and functions. These enzymes reduce hydrogen peroxide, organic hydroperoxides, and peroxynitrite using a conserved peroxidatic cysteine that becomes oxidized and is subsequently recycled back to its active form—typically by thioredoxin (Trx), although some isoforms utilize other reducing agents [10,11]. The 2-Cys Prxs (Prx1–4) function as homodimers, while Prx5 is an atypical 2-Cys Prx that acts as a monomer, and Prx6 is the only mammalian 1-Cys Prx that lacks a resolving cysteine. Beyond peroxide detoxification, Prxs also participate in redox signaling, inflammation, and cell survival [12]. Prxs are regulated at the transcriptional level (e.g., by nuclear factor erythroid 2-related factor 2 (Nrf2), a master regulator of antioxidant mechanisms) [13]), but also by posttranslational modifications such as phosphorylation and acetylation [14].

Prxs are widely expressed throughout the brain (https://www.proteinatlas.org, accessed on 1 August 2025). However, the expression of individual Prxs varies by cell type. For example, Prx2 is expressed at much higher levels in neurons than astrocytes, whereas the reverse occurs for Prx6 (https://www.proteinatlas.org, accessed on 1 August 2025). Evidence indicates that Prxs have both physiological functions and roles in pathological conditions, including hemorrhagic and ischemic stroke, where their dysregulation may contribute to disease progression and represent potential therapeutic targets. However, the underlying mechanisms of action for Prxs in stroke have largely remained elusive. Therefore, this review aims to elucidate the significance of Prxs in stroke, contributing to a deeper understanding of their potential value as novel therapeutic targets. The most relevant studies on this topic were identified through MEDLINE (accessed by PubMed on 16 June 2025) using the following terms: “peroxiredoxin”, or “Prx”, or “Prdx” and “hemorrhagic stroke”, or “intracerebral hemorrhage”, or “intracranial hemorrhage”, or “intraparenchymal hemorrhage” and “subarachnoid hemorrhage”, and “intraventricular hemorrhage”, and “ischemic stroke”, or “stroke”. The reference lists for each article were reviewed to search for additional relevant studies. Both preclinical and clinical studies were included if they reported effects and mechanisms of action for Prxs in stroke.

## 2. Prxs and Hemorrhagic Stroke

Prxs are redox-regulating enzymes with context-dependent roles in hemorrhagic stroke. Their expression and function vary by isoform, cell type, and timing, influencing oxidative stress, inflammation, and neuronal survival across different forms of hemorrhagic stroke.

### 2.1. Prx1 and Intracerebral Hemorrhage

Prx1 exhibits dynamic and situation-specific roles in ICH, functioning as both a proinflammatory mediator and a neuroprotective regulator depending on timing, localization, and cell type. Several studies [15,16,17,18] report that Prx1 expression is significantly upregulated in the perihematomal region after ICH, particularly in astrocytes and microglia, peaking between 72 h and 7 days post-injury. In the acute phase, Prx1 acts as a damage-associated molecular pattern (DAMP) molecule, promoting neuroinflammation through TLR4/NF-κB signaling and triggering the release of nitric oxide (NO), interleukin 6 (IL-6), and tumor necrosis factor alpha (TNF-α), thereby contributing to secondary brain injury [16]. However, conflicting findings [19] suggest that Prx1 is not always extracellular; in some cases, it remains nuclear within apoptotic cells, indicating different mechanistic roles depending on injury severity and cellular fate.

Interestingly, overexpression of Prx1 or its upregulation after injection of Danhong (a Chinese herbal medicine) has shown protective effects, including reduced brain edema, hematoma volume, and neuronal apoptosis, alongside improved neurological outcomes [17]. This neuroprotection appears to be astrocyte-dependent, as inhibiting Prx1 abrogated the beneficial effects of Danhong. Moreover, ribonucleic acid (RNA) immunoprecipitation data demonstrate that Prx1 may function as an RNA-binding protein, stabilizing transcripts involved in anti-apoptotic and anti-inflammatory signaling, such as BCL6 and PTEN [18]. These findings reveal a dual role: Prx1 can initiate inflammation early on but also contributes to repair and homeostasis in later stages.

Additionally, co-expression of heme oxygenase-1 (HO-1) and Prx1 in reactive astrocytes and microglia suggests a coordinated antioxidative response, where HO-1 may preserve Prx1 activity by degrading heme, which otherwise inhibits Prx1 [15]. It is important to note that HO-1 is a prototypical oxidative stress-responsive gene that is often induced via redox-sensitive transcriptional programs (e.g., Nrf2/antioxidant response elements [20]. Prxs, including Prx1, can likewise be regulated at the transcriptional level in response to oxidative and inflammatory cues, in addition to post-translational modifications that shape their localization and function [21,22]. The therapeutic potential of targeting Prx1 is further supported by findings that two phytochemicals, ligustilide and senkyunolide, reduce Prx1 expression and neuronal apoptosis [19]. In sum, Prx1 plays both detrimental and protective roles in ICH, with its effects shaped by spatiotemporal dynamics, post-translational regulation, and interactions with pathways such as HO-1 and TLR4/NF-κB. Effective therapeutic strategies may need to modulate rather than suppress Prx1, enhancing its protective actions while minimizing its inflammatory effects.

### 2.2. Prx2 and Intracerebral Hemorrhage

**Figure 1:**
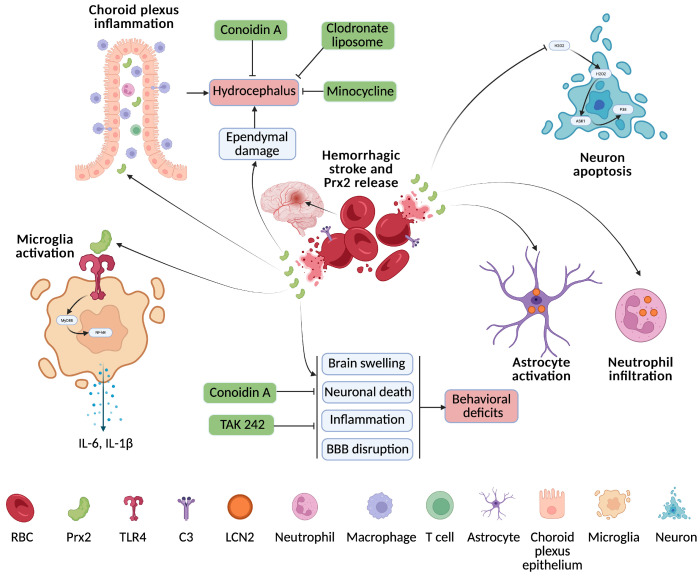
Prx2 released from red blood cells after a hemorrhagic stroke causes brain injury via multiple pathways. Complement C3 is a key upstream regulator of Prx2 release. Prx2 strongly induces LCN2 expression in neutrophils and astrocytes, activates microglia, and enhances IL-1β and IL-6 production via the TLR4/MyD88/NF-κB pathway. Inhibiting Prx2 worsens outcomes by increasing oxidative stress and apoptosis through the H_2_O_2_–ASK1/p38 pathway. Prx2 leads to brain swelling, neuronal death, inflammation, and behavioral deficits, which are attenuated by Conoidin A and TAK242. It also increases macrophages, T cells, and neutrophils in the choroid plexus, causing ependymal damage and hydrocephalus—effects reduced by Conoidin A, minocycline, and clodronate liposomes.

There has been a particular interest in the role of Prx2 in cerebral hemorrhage because it is the third most common protein in erythrocytes [23]. While it plays an essential role in protecting those cells from oxidative stress [24], the release of Prx2 into the extracellular space after erythrolysis may have an important role in brain injury (Figure 1). Thus, emerging evidence has established Prx2 as a key mediator in the pathophysiology of ICH, with both detrimental and protective roles depending on its cellular context and signaling. Our group’s study [25] demonstrated that lysed red blood cells significantly increased brain Prx2 levels in rats, resulting in brain swelling, neuronal death, inflammation, and behavioral deficits. These detrimental effects were attenuated by co-injection with the Prx2 inhibitor conoidin A, which reduced tissue damage and improved neurological outcomes. Consistent with these findings, another study [26] reported a marked rise in Prx2 levels following ICH in mice, where exogenous Prx2 exacerbated BBB disruption and immune cell activation, further impairing neurological function. Notably, both studies highlight the pathological role of Prx2 in ICH and demonstrate that targeting Prx2—either directly or via TLR4 inhibition (TAK 242)—can mitigate neuroinflammation and promote recovery, underscoring its potential as a therapeutic target.

Recent studies have begun to uncover the molecular mechanisms underlying Prx2-mediated neurotoxicity following ICH, highlighting its upstream regulators and downstream effectors. Complement component C3 has been identified as a key upstream regulator of Prx2 release from erythrocytes, linking innate immune activation with Prx2-mediated brain injury [27]. Peripheral immune cell recruitment is also a prominent feature of Prx2’s action. Prx2 promotes infiltration of monocyte-derived macrophages and activation of microglia, expanding the inflammatory cascade within the injured brain [28]. The role of lipocalin-2 (LCN-2) as a downstream effector of Prx2 has been confirmed, with its expression strongly induced by Prx2 in neutrophils and astrocytes [29]. LCN-2 deficiency protects against Prx2-induced neuronal damage and functional impairment, indicating its critical role in mediating Prx2 toxicity.

A clinical study [30] found that serum Prx2 levels rise significantly after ICH and correlate with stroke severity and poor outcomes. Elevated Prx2 independently predicted early neurological deterioration, stroke-associated pneumonia, and 6-month prognosis, making it a promising biomarker for assessing ICH severity and clinical progression. However, one in vitro study [31] found that Prx2 protects neurons from pyroptosis after ICH by inhibiting MAPK-NF-κB signaling, preserving mitochondrial function, and reducing ER stress via the PI3K/AKT pathway. Knockdown of Prx2 exacerbates pyroptosis and neuronal injury, highlighting a critical neuroprotective role.

Taken together, these findings underscore the variable, condition-sensitive role of Prx2 in ICH. While extracellular or excessively activated Prx2 promotes inflammation and injury, its intracellular function may be essential for neuronal survival, highlighting the importance of selective targeting strategies in future therapies.

### 2.3. Prx3 and Intracerebral Hemorrhage

A study [32] found that the expression of Thioredoxin-reductase 2 (Txnrd2), Trx2, and Prx3 was significantly upregulated in neurons and astrocytes following ICH, peaking around 48–72 h. Silencing Txnrd2 via small interfering RNA (siRNA) aggravated neurological deficits, brain edema, oxidative stress, and ER stress, accompanied by decreased levels of Trx2 and Prx3. Selenium treatment post-ICH reduced neuronal injury, brain edema, and neurological deficits, and effectively restored Txnrd2, Trx2, and Prx3 expression. Mechanistically, selenium alleviated oxidative and ER stress by upregulating Txnrd2, suggesting a protective role of the Txnrd2/Trx2/Prx3 axis in ICH pathology.

### 2.4. Prx6 and Intracerebral Hemorrhage

A proteomic analysis of the cerebral cortex from *Sus scrofa* (porcine) following hemorrhagic stroke revealed a significant increase in the relative abundance of Prx6 24 h after injury [33]. Prx6 was among a group of extracellular and exosomal proteins associated with neuroprotective functions, including oxidative stress compensation and apoptosis regulation. This elevation suggests that Prx6 may be involved in early neuroprotective responses after ICH.

### 2.5. Prx1/2 and Subarachnoid Hemorrhage

One study reported that elevated levels of Prx2 in the cerebral cortex and cerebrospinal fluid (CSF) following SAH may result from its release from lysed erythrocytes [34]. Similarly, Connor et al. [35] identified increased Prx2 expression in the CSF after SAH through a proteomic analysis of patient samples. After SAH, Prx1 and Prx2 are rapidly upregulated—Prx1 in astrocytes peaking at 6 h, and Prx2 in neurons peaking at 12 h. Inhibiting Prx1/2 worsens neurological outcomes by enhancing oxidative stress and apoptosis via the H_2_O_2_–ASK1/p38 pathway, while their overexpression offers neuroprotection by reducing oxidative damage and inflammation [36] (Figure 1). However, interestingly, after SAH, Prx2 promotes neuronal injury by activating microglia and enhancing their proinflammatory responses, including increased IL-1β and IL-6 production via the TLR4/MyD88/NF-κB pathway [37]. Inhibition of this signaling cascade significantly attenuates Prx2-induced neurotoxicity. Clinically, elevated CSF [37] and serum [38] Prx2 levels in SAH patients correlate with greater neurological severity, as measured by Hunt-Hess, World Federation of Neurosurgical Societies (WFNS), and mFisher scores. Higher serum Prx2 is also independently associated with poorer 6-month outcomes and increased risk of delayed cerebral ischemia. Unlike the intracellular antioxidant roles seen in animal models, extracellular Prx2 in patients may drive pathology by promoting inflammation. Thus, Prx2 has dual and context-governed roles—intracellularly protective yet extracellularly proinflammatory—making them both potential therapeutic targets and prognostic biomarkers in SAH.

### 2.6. Prx3/5 and Subarachnoid Hemorrhage

Prx3, a mitochondria-localized antioxidant protein, plays a protective role in early brain injury following SAH [39]. After SAH, endogenous neuronal Prx3 levels significantly decrease, contributing to elevated hydrogen peroxide levels and neuronal apoptosis. Overexpression of Prx3 via neuron-specific adeno-associated virus delivery restores Prx3 levels, reduces oxidative stress and caspase-9-mediated apoptosis, and enhances neuronal survival. This intervention also improves both short- and long-term neurological outcomes, including sensorimotor coordination and spatial memory. Another study [40] demonstrates that Prx5 plays a neuroprotective role in early brain injury following SAH by reducing oxidative stress and neuronal apoptosis. Endogenous Prx5 expression significantly decreases post-SAH, and intranasal administration of recombinant Prx5 improves both short- and long-term neurological outcomes, reduces brain edema, and preserves BBB integrity. Recombinant Prx5 also restores antioxidant enzyme activity, though the underlying mechanism—whether through increased expression or enhanced enzymatic function—remains to be fully elucidated. Recombinant Prx5 also restores antioxidant enzyme activity and upregulates anti-apoptotic proteins (Bcl-2, Bcl-xl) while suppressing pro-apoptotic markers (Bax, Cleaved Caspase-3). Prx5 knockdown using siRNA reverses these protective effects, exacerbating oxidative stress, apoptosis, and functional deficits. These findings suggest that Prx5 is a promising therapeutic target for mitigating early brain injury after SAH.

### 2.7. Prx2 and Intraventricular Hemorrhage

Following intraventricular injection of Prx2 in mice, there was a significant increase in lateral ventricle size and choroid plexus (ChP) macrophages—especially epiplexus macrophages—compared to saline controls. Additionally, both CD4(+) T lymphocytes and neutrophils in the ChP were markedly elevated after Prx2 exposure [41]. These findings suggest that Prx2 contributes to immune activation at the ChP after IVH, potentially exacerbating inflammation and causing hydrocephalus. Following IVH in a rat model, Prx2 levels rise significantly in the periventricular zone, and exogenous Prx2 injection induces ventricular dilation—particularly in male rats—along with ependymal disruption and ventricle wall damage [42]. Prx2 triggers immune activation in the ChP, increasing OX-6(+) macrophages, especially epiplexus cells, and shifting their phenotype toward a proinflammatory profile with more OX-6(+)/Iba-1(+) macrophages and fewer dendritic cells. This is accompanied by increased infiltration of inflammatory cells, including myeloperoxidase-, Iba-1-, and CD68-positive populations [43]. Pharmacologic inhibition of Prx2 with conoidin A, as well as ChP macrophage depletion via clodronate liposomes or treatment with minocycline, significantly reduces Prx2-induced inflammation, immune cell activation, and ventricular damage (Figure 1). These findings highlight Prx2 as a key mediator of post-hemorrhagic hydrocephalus and ChP inflammation. They support its potential as a therapeutic target.

In summary, Prx1 and Prx2 play dual roles in ICH, promoting inflammation in the acute phase through TLR4/NF-κB signaling but also exhibiting neuroprotective effects in later stages by stabilizing anti-apoptotic transcripts or inhibiting pyroptosis. In SAH, Prx1 and Prx2 are rapidly upregulated, and while intracellular Prx2 is protective, extracellular Prx2 contributes to microglial activation and inflammation, correlating with worse clinical outcomes. Prx3 and Prx5 are mitochondrial antioxidants that reduce oxidative stress and apoptosis after SAH, improving both short- and long-term neurological outcomes, with Prx5 also preserving BBB integrity. In IVH, Prx2 exacerbates inflammation and hydrocephalus via immune activation in the choroid plexus, effects that are reversible with pharmacologic inhibition or macrophage depletion. Prx6, identified in porcine models of ICH, may participate in early neuroprotective responses through exosomal antioxidant signaling. Together, these findings highlight the isoform- and context-specific actions of Prxs, emphasizing their potential as therapeutic targets and biomarkers in hemorrhagic stroke.

## 3. Prxs and Ischemic Stroke

Prxs are redox-regulating enzymes with context-specific roles in ischemic stroke, functioning both as intracellular antioxidants and extracellular proinflammatory mediators. Their impact depends on isoform, localization, cellular source, and post-translational modifications, making them complex yet promising targets in stroke therapy.

### 3.1. Prx1 and Ischemic Stroke

Prx1 plays a complex, dual role in ischemic stroke pathology, functioning as both a neuroprotective antioxidant and a DAMP that exacerbates inflammation. Multiple studies have identified Prx1 as a key inflammatory mediator after stroke. It is released from necrotic brain cells and activates microglia via the TLR4/NF-κB pathway, contributing to neuroinflammation and secondary injury [44,45,46]. Among Prx family members, Prx1 is one of the most prominent in inducing IL-23 and downstream IL-17 signaling, amplifying post-ischemic immune responses [44]. Blocking Prx1 or using Prx1-targeted peptides effectively reduces infarct size, neuronal apoptosis, and behavioral deficits, indicating therapeutic potential [46]. Delayed administration of the poly (ADP-ribose) polymerase 1 (PARP-1) inhibitor PJ34 downregulates Prx1 and improves outcomes after middle cerebral artery occlusion (MCAO), particularly in males, suggesting sex-dependent regulatory effects [45]. The proinflammatory role of Prx1 is further demonstrated by its ability to reverse the anti-inflammatory benefits of PJ34 when administered exogenously. Similarly, Gas-D, a derivative of the natural compound gastrodin from some orchids, reduces stroke-induced injury by attenuating Prx1-induced inflammation via TLR4/NF-κB inhibition, emphasizing the relevance of targeting extracellular Prx1 [47].

However, intracellular Prx1 also serves antioxidant and cytoprotective functions. After ischemic injury, Prx1 is upregulated in endothelial cells, and it mitigates BBB disruption and neuronal apoptosis. Yet, this protective role is compromised by E6AP-mediated ubiquitination during prolonged ischemia [48]. Enhancing Prx1 levels via lentiviral delivery improves neurological outcomes and preserves BBB integrity [48], suggesting a time- and location-dependent role for Prx1 in stroke. Recent work identified a specialized stroke-associated microglia (SAM) population dependent on Prx1 for activation and survival. These cells express antioxidant pathways like Prx1-Srxn1-Txn1 and offer neuroprotection through oxidative stress reduction [49]. In Prx1-deficient mice, reduced SAM formation leads to larger infarcts and worse outcomes, again indicating a role of Prx1 in protective microglial adaptation.

Ischemic postconditioning downregulates Prx1, which otherwise promotes macrophage-mediated inflammation and immune checkpoint marker expression [50]. These findings suggest that modulating Prx1 might enhance recovery by reducing sustained immune activation. However, Prx1 may not serve as a useful biomarker for stroke recurrence; a study found no correlation between plasma Prx1 levels and recurrent cerebrovascular events, whereas high-sensitivity C-Reactive Protein (hs-CRP) was predictive [51]. Interestingly, in genetic studies, carriers of deleterious variants in the protein Z (PROZ) gene exhibited elevated plasma Prx1 levels and higher ischemic stroke risk, suggesting a link between PROZ dysfunction and Prx1-driven oxidative responses [52]. Finally, CD21 improves outcomes in tissue plasminogen activator (tPA)-treated stroke by promoting clearance of extracellular Prx1 via MSR1 in M2 macrophages. This not only suppresses neuroinflammation but also preserves BBB integrity, confirming that efficient Prx1 removal is critical in managing post-stroke inflammation [53].

Prx1 exerts both beneficial and harmful effects in ischemic stroke depending on its location (intracellular vs. extracellular), cellular source (endothelium, microglia, necrotic neurons), and timing. Therapeutic strategies aiming to enhance intracellular Prx1 antioxidant function while neutralizing or clearing extracellular Prx1 may offer the most balanced and effective approach for reducing ischemic brain injury.

### 3.2. Prx2 and Ischemic Stroke

**Figure 2:**
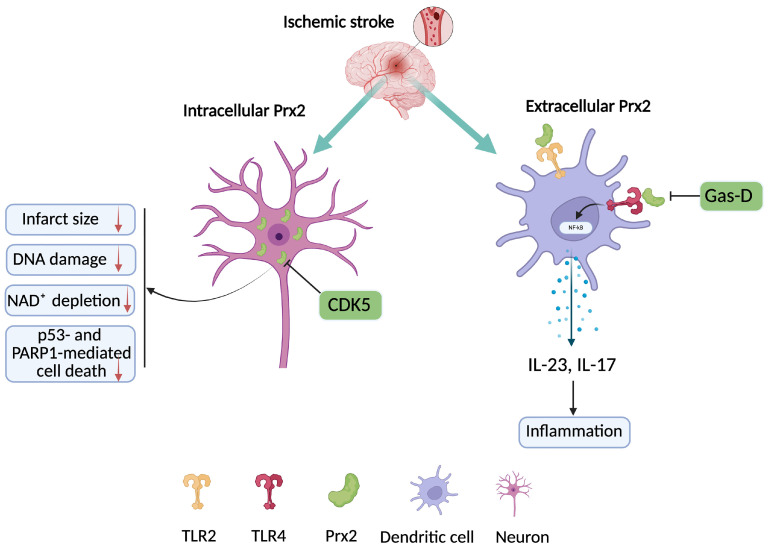
Neuronal overexpression of intracellular Prx2 reduces infarct size, DNA damage, and NAD^+^ depletion, and inhibits p53/PARP1-mediated cell death. CDK5 phosphorylates Prx2 at threonine 89, inactivating it and promoting neuronal death. Extracellular Prx2 triggers inflammation by activating dendritic cells via TLR2/4 and inducing IL-23/IL-17 signaling. Gas-D suppresses Prx2-induced macrophage activation by inhibiting the TLR4/NF-κB pathway.

Prx2 plays a multifaceted role in the pathophysiology of ischemic stroke, acting as both a critical endogenous antioxidant and a proinflammatory DAMP depending on its context, location, and post-translational modifications (Figure 2).

Early after ischemic stroke, Prx2 is rapidly upregulated in the ischemic hemisphere [54], suggesting an acute neuroprotective response against oxidative stress. This is supported by findings that overexpression of Prx2 in neurons reduces infarct size, DNA damage, NAD^+^ depletion, and suppresses p53- and PARP1-mediated cell death, highlighting its essential role in maintaining redox homeostasis and genomic integrity after ischemia. Notably, knockdown of Prx2—but not Prx1—exacerbated neuronal injury in oxygen-glucose deprivation (OGD) models, indicating Prx2’s more prominent role in neuronal antioxidant defense [55]. However, ischemia-induced post-translational modifications of Prx2 can transform its function. One study found that cyclin-dependent kinase 5 (CDK5) phosphorylates Prx2 at threonine, rendering it inactive and promoting neuronal death role [56]. Overexpression of a non-phosphorylatable mutant Prx2T89A was neuroprotective, whereas a phospho-mimic failed to protect neurons, confirming that CDK5-mediated phosphorylation disrupts Prx2’s antioxidant role. These findings suggest that post-ischemic modifications of Prx2 determine its fate as either protective or harmful.

In diabetic conditions, Prx2 expression is significantly reduced after ischemic insult, which may explain the larger infarct volumes and exacerbated injury observed in diabetic stroke models [57]. This supports the concept that baseline redox balance and Prx2 availability are critical for stroke outcome, and impaired Prx2 signaling in diabetes compromises neuronal resilience. Conversely, Prx2 can also act as an extracellular DAMP once released from necrotic brain cells. It contributes to inflammation by activating dendritic cells through TLR2 and TLR4 pathways and promoting IL-23 and IL-17 signaling cascades, thus linking oxidative injury to immune activation [44]. These proinflammatory effects are independent of Prx2’s antioxidant activity, indicating that its extracellular form may be deleterious post-stroke. Targeting this dual nature of Prx2, the compound Gas-D has been shown to suppress Prx2-induced macrophage activation by inhibiting the TLR4/NF-κB pathway (Figure 2), reducing cytokine production and infarct size even when administered 10 h post-stroke [47]. Gas-D not only inhibits TLR signaling but also reduces the extracellular release of Prx2 itself, positioning it as a promising therapeutic for modulating Prx2-mediated damage.

Interestingly, the differential temporal expression of Prxs after stroke further illustrates Prx2’s unique role. While Prx2 and Prx5 are acutely induced at 24 h, Prx1 and Prx4 peak later, suggesting Prx2’s involvement in early redox responses, whereas others may participate in subacute inflammation or repair [54]. Collectively, these studies depict Prx2 as a double-edged sword: intracellular Prx2 protects neurons through ROS scavenging and energy preservation, while extracellular or modified Prx2 can propagate inflammation and injury. Therapeutic approaches should aim to preserve or enhance the endogenous, cytoplasmic antioxidant function of Prx2 while preventing its extracellular release or pathological modification. This dual-targeting strategy could offer precise neuroprotection in the complex landscape of ischemic stroke.

### 3.3. Prx4 and Ischemic Stroke

Prx4 plays a crucial neuroprotective role in ischemic stroke, particularly by preserving BBB integrity and reducing neuroinflammation. Endogenously, Prx4 is markedly upregulated in brain endothelial cells post-stroke, where it inhibits myosin light chain phosphorylation to prevent cytoskeletal stress and tight junction disruption, thereby limiting BBB leakage and subsequent immune cell infiltration [58]. Loss of endothelial Prx4 worsens infarct size and long-term outcomes, underscoring its intrinsic protective role. Exogenously, Prx4 secreted by CCR2-engineered mesenchymal stem cells (MSC^CCR2) also contributes significantly to stroke recovery by attenuating oxidative stress, stabilizing endothelial junctions, and reducing inflammatory responses [59]. Importantly, knockdown of Prx4 in MSC^CCR2 nullifies their therapeutic effects, further validating the central function of Prx4. These studies highlight the dual capacity of Prx4: as an endogenous endothelial antioxidant and as a therapeutic effector delivered by stem cells. Together, these findings suggest that targeting or enhancing Prx4 activity offers a promising strategy to protect the neurovascular unit and improve functional recovery after ischemic stroke.

### 3.4. Prx5 and Ischemic Stroke

Prx5 appears to play a protective and potentially anti-inflammatory role in ischemic stroke, although its clinical significance remains nuanced. In stroke patients, lower plasma Prx5 levels were associated with more severe strokes and higher systemic inflammation, suggesting that Prx5 may act as an endogenous anti-inflammatory factor [60]. However, Prx5 levels measured on day 3 post-stroke did not independently predict long-term outcomes, indicating that while Prx5 reflects early stroke severity, it may not serve as a reliable prognostic biomarker. In contrast, animal studies revealed that cerebral ischemia led to significant downregulation of Prx5 expression in the cortex, an effect prevented by treatment with resveratrol [61]. Resveratrol maintained both protein and mRNA levels of Prx5 at near-normal levels, correlating with improved neuroprotection and suggesting that preserving Prx5 expression may help limit oxidative damage. Together, these findings imply that Prx5 may mitigate ischemia-induced injury through antioxidant and anti-inflammatory mechanisms, and that therapeutic strategies like resveratrol could enhance its protective effects. Thus, while Prx5 may not predict outcomes, its modulation could offer a therapeutic avenue for reducing ischemic brain injury.

### 3.5. Prx6 and Ischemic Stroke

Prx6 exhibits multifaceted and condition-dependent roles in ischemic stroke, acting as both a neuroprotective antioxidant and a proinflammatory mediator depending on its cellular source, localization, and post-translational modifications. Several studies highlight the neuroprotective function of intracellular Prx6, particularly when upregulated in neurons. For instance, 4-hydroxybenzoic acid enhances neuronal survival in MCAO models by activating PI3K/Akt signaling, which promotes nuclear translocation of Nrf2 and subsequent Prx6 expression; this effect is abolished by phosphatidylinositol 3-kinase (PI3K) inhibition, confirming the pathway’s specificity [62]. Additionally, extracts of *Gastrodia elata* Blume and its active compound 4-hydroxybenzyl alcohol boost Prx6 expression and offer neuroprotection, implying that modulation of different Prx isoforms contributes to antioxidative stroke defense [63]. Similarly, curcumin confers neuroprotection through SP1-mediated upregulation of Prx6 in the peri-infarct cortex, leading to reduced oxidative stress and infarct size—effects reversed by Prx6 knockdown [64]. Supporting these findings, Schwann-like cell (SCLC)-conditioned medium and recombinant Prx6 protein alleviate neuronal apoptosis and oxidative damage via PTEN/PI3K/AKT signaling, while neutralizing Prx6 abolishes these therapeutic effects [65]. Prx6 knockdown studies [66] further underscore its protective capacity: silencing Prx6 in rats worsens ischemia–reperfusion injury, increases infarct volume, and promotes neuronal apoptosis and mitophagy via PTEN-induced kinase 1 (PINK1)-mediated pathways, while co-knockdown of Prx6 and PINK1 reverses these detrimental effects. One study [67] found that Prx6 may also be implicated in the antioxidant effects of melatonin on rat ischemic stroke. These results collectively position intracellular Prx6 as a critical mediator of antioxidant defense and mitochondrial homeostasis.

However, not all roles of Prx6 are beneficial. Under ischemic conditions, astrocytes subjected to OGD and reoxygenation release Prx6 extracellularly, and this release—peaking at 24 h—is dependent on its acidic, Ca^2+^-independent, phospholipase A2 (aiPLA2) activity [68]. Extracellular Prx6 promotes neuroapoptosis via the RAGE/JNK pathway, and blocking the receptor for advanced glycation end products (RAGE) mitigates infarct size and neurological damage, highlighting this pathway as a therapeutic target. Similarly, a study [69] highlights that the aiPLA2 activity of Prx6 is responsible for TLR2/4-mediated inflammatory brain injury and the release of neurotoxic mediators (Nuclear factor kappa-light-chain-enhancer of activated B cells (NF-κB), inducible nitric oxide synthase (iNOS), and cyclooxygenase-2 (COX-2)) in a rat model of MCAO. Targeting the aiLA2 activity of Prx6 with the siRNA or pharmacological inhibitors has shown a diminution in neurologic deficits, cerebral infarction, brain water content, and inflammatory molecules. Moreover, astrocytic Prx6-aiPLA2 contributes to microglial activation and polarization toward a proinflammatory phenotype, mediated by ROS production and mitochondrial fission; inhibiting this activity via MJ33 reduces infarct size and inflammation [70].

Further evidence from Tet methylcytosine dioxygenase 1 (TET-1) mice, which overexpress endothelial endothelin 1, reveals that these mice exhibit more severe neurovascular injury and behavioral deficits, alongside increased astrocytic Prx6 expression near blood vessels and enhanced caspase-3 levels, suggesting a detrimental astrocytic Prx6 contribution under chronic stress [71]. Prior to MCAO, administering human cerebral endothelial cells into the left common carotid artery of rats can suppress Prx6 expression [72]. Moreover, a natural product, ligustilide, provides neuroprotection by suppressing both intracellular expression and extracellular release of Prx6, shifting immune responses toward anti-inflammatory profiles and reducing neurodegeneration through inhibition of TLR4, p-ERK, p-STAT3, and NF-κB signaling [73].

Taken together, these studies reveal a location- and context-specific role for Prx6: its intracellular neuronal expression supports antioxidant defenses and mitochondrial regulation, whereas its extracellular or astrocytic release—especially via aiPLA2 activity—amplifies oxidative stress and neuroinflammation. Therapeutic strategies for ischemic stroke should therefore consider enhancing intracellular Prx6 function while suppressing its extracellular release and enzymatic activation in astrocytes to maximize neuroprotection.

In summary, Prx1 acts as a double-edged sword in ischemic stroke, promoting inflammation via TLR4/NF-κB when extracellular, yet offering neuroprotection intracellularly by preserving the BBB and supporting specialized microglial subpopulations. Prx2 provides robust antioxidant defense early post-stroke, but can become harmful when phosphorylated or released extracellularly, triggering immune activation through TLR2/4 signaling. Prx4 protects vascular integrity by stabilizing endothelial tight junctions and reducing inflammation, functioning both endogenously and through therapeutic stem cell delivery. Prx5 appears to suppress inflammation and oxidative stress, and its levels correlate with stroke severity, though not long-term prognosis. Prx6 exhibits a dual role: intracellularly, it supports neuronal survival and mitochondrial function; extracellularly, especially from astrocytes, it exacerbates inflammation and neuronal injury via aiPLA2-dependent pathways. Altogether, therapeutic approaches should selectively enhance protective intracellular Prx functions while inhibiting their harmful extracellular or modified forms to optimize stroke outcomes.

## 4. Comparisons of the Roles of Prxs in Hemorrhagic and Ischemic Stroke

The Prx family members—Prx1 through Prx6—demonstrate both shared and divergent roles in hemorrhagic and ischemic stroke, with isoform-specific expression patterns, situation-specific functions, and differential cellular localization determining their impact on brain injury and recovery.

Prx1 plays a dual and context-dependent role in both hemorrhagic and ischemic stroke, acting as a proinflammatory mediator when extracellular and a neuroprotective antioxidant when intracellular. In hemorrhagic stroke, especially ICH, Prx1 is upregulated in astrocytes and microglia, triggering TLR4/NF-κB-mediated inflammation in the acute phase but later supporting anti-apoptotic signaling and neuroprotection. Similarly, in ischemic stroke, extracellular Prx1 amplifies immune responses via IL-23/IL-17 pathways, while intracellular Prx1—particularly in endothelial cells and stroke-associated microglia—helps preserve BBB integrity and reduce injury. Ischemic models further reveal post-translational regulation of Prx1, such as E6AP-mediated degradation, which impairs its protective effects.

Prx2 exhibits context-specific, dual functions in both hemorrhagic and ischemic stroke. In hemorrhagic stroke, particularly ICH, SAH, and IVH, extracellular Prx2 acts as a DAMP that exacerbates neuroinflammation via TLR4/MyD88/NF-κB signaling, promoting immune cell infiltration, cytokine release, and neuronal damage. It is also a biomarker of severity in clinical settings. Conversely, intracellular Prx2 may play protective roles, reducing oxidative stress and neuronal death. Similarly, in ischemic stroke, intracellular Prx2 protects neurons from oxidative damage by maintaining redox balance and suppressing cell death pathways, while its extracellular or phosphorylated form promotes inflammation through TLR2/4 and CDK5-mediated inactivation. Thus, across both stroke types, Prx2 demonstrates a clear dichotomy: neuroprotective when intracellular and deleterious when extracellular or pathologically modified. A major difference between hemorrhagic and ischemic stroke is the role of erythrocyte Prx2 in the former. The very high concentrations of Prx2 in those cells make preventing its release an attractive therapeutic target.

Prx3, primarily a mitochondrial antioxidant, plays a protective role in ICH by maintaining redox homeostasis and reducing ER stress through the Txnrd2/Trx2 axis. Its upregulation by selenium improves neurological outcomes. Prx3 also plays a protective role in early brain injury following SAH by reducing oxidative stress and caspase-9-mediated apoptosis, thereby enhancing neuronal survival. In contrast, the effects of Prx3 are not well-characterized in ischemic stroke.

Prx4 is predominantly protective in ischemic stroke. It is upregulated in endothelial cells, where it preserves BBB integrity and limits immune cell infiltration. Secreted Prx4 from mesenchymal stem cells enhances recovery by stabilizing vascular function. The role of Prx4 in hemorrhagic stroke remains poorly defined, indicating a potential area for future research.

Prx5 demonstrates neuroprotective effects in hemorrhagic stroke, particularly in SAH, where its recombinant form reduces oxidative stress, apoptosis, and BBB disruption while improving neurological outcomes. In ischemic stroke, Prx5 is downregulated following injury, and its preservation via compounds like resveratrol is associated with improved neuroprotection. Clinically, lower plasma Prx5 levels correlate with higher stroke severity, suggesting an anti-inflammatory role. Thus, Prx5 acts as an antioxidant and anti-apoptotic agent in both hemorrhagic and ischemic stroke, although its prognostic utility appears limited to early-stage severity assessments

Prx6 has contrasting roles depending on its localization. In hemorrhagic stroke (e.g., ICH in porcine models), elevated Prx6 levels are associated with early neuroprotective responses, possibly through exosomal antioxidant signaling. In ischemic stroke, intracellular Prx6 confers robust neuroprotection by enhancing PI3K/AKT/Nrf2 pathways, reducing oxidative stress, and supporting mitochondrial integrity. However, extracellular Prx6—especially from astrocytes—amplifies inflammation via aiPLA2 activity, TLR signaling, and RAGE/JNK pathways, worsening injury. Therefore, Prx6 displays protective effects intracellularly across both stroke types but promotes neuroinflammation when released extracellularly during ischemia, underscoring the importance of targeting its source and form in therapeutic strategies.

In summary, all Prx isoforms exhibit dynamic and often contrasting roles in stroke depending on their cellular location, timing, and post-translational modifications. Prx1 and Prx2 are prominent DAMPs and inflammatory mediators when extracellular, while serving antioxidant and neuroprotective roles intracellularly. Prx3, Prx4, and Prx5 mainly offer protection, with Prx4 and Prx5 more relevant in ischemia. The function of Prx6 is highly dependent on its source and enzymatic activity. Isoform- and context-specific modulation of Prxs may offer therapeutic strategies for minimizing damage and enhancing recovery after both hemorrhagic and ischemic stroke.

## 5. Future Directions

Despite significant advances, several critical questions remain regarding the precise roles and regulatory mechanisms of Prx isoforms in stroke. Key unresolved issues include the factors that govern the switch between the protective and pathological roles of specific Prxs, particularly in relation to their subcellular localization and post-translational modifications. Intracellular versus extracellular Prx activity likely reflects two dynamically regulated pools rather than fixed “forms”. These dynamics may shift the balance between cytoprotective and proinflammatory effects over time and across brain cell types after stroke. However, quantitative, isoform-resolved in vivo data directly linking intracellular post-translational modification states to extracellular abundance and biochemical speciation remain limited, and addressing this gap is an important direction for future work. Additionally, systemic variables such as age, sex, and comorbidities (e.g., diabetes and hypertension) may influence Prx expression and function, but their exact impact remains unclear. Meanwhile, the long-term consequences of modulating Prx signaling on post-stroke recovery and neural plasticity also warrant further investigation. While acute neuroinflammation after stroke may enhance brain injury, neuroinflammation is also an important component of long-term brain repair and, in hemorrhagic stroke, hematoma resolution.

Emerging tools such as single-cell transcriptomics and spatial proteomics offer high-resolution insights into cell-type-specific Prx dynamics [74,75], while clustered regularly interspaced short palindromic repeats (CRISPR)-based gene editing [76] and isoform-specific inhibitors—such as those targeting the aiPLA2 activity of Prx6—provide precise strategies for dissecting Prx function. Advanced imaging technologies may also enable real-time tracking of redox changes and extracellular Prx release in vivo [77]. Furthermore, cell-based delivery systems—such as mesenchymal stem cells engineered to secrete protective Prx isoforms—represent an innovative and targeted approach to stroke therapy [78,79]. From a translational perspective, Prx isoforms, especially Prx2 and Prx1, are promising as diagnostic and prognostic biomarkers due to their detectable levels in CSF and serum following stroke. Pharmacological modulation of Prxs in stroke is summarized in Table 1, and genetic approaches to studying Prxs in stroke are summarized in Table 2. Therapeutic strategies that combine the enhancement of intracellular Prx activity with the neutralization of extracellular Prx-driven inflammation may yield synergistic neuroprotective effects. Ultimately, a deeper mechanistic understanding of the multifaceted and variable role depending on cellular and temporal context of Prxs will be critical for harnessing their full therapeutic potential in stroke and other neuroinflammatory conditions. BBB permeable specific inhibitors and enhancers of different Prx isoforms would aid greatly in such studies.

## 6. Summary

Stroke, encompassing both ischemic and hemorrhagic subtypes, triggers a cascade of oxidative stress and neuroinflammation that contributes to secondary brain injury and neurological deficits. The Prx (Prdx) family—six thiol-dependent antioxidant enzymes—emerges as a central player in this process, bridging redox regulation with immune signaling. This review synthesizes recent evidence on the isoform-specific roles of Prxs across stroke types, revealing their roles that vary with pathological conditions. While intracellular Prxs often mitigate oxidative damage and support neuroprotection, their extracellular counterparts—particularly Prx1 and Prx2—can act as proinflammatory DAMPs, exacerbating brain injury through toll-like receptor pathways. Isoforms like Prx3 and Prx4 show protective roles via mitochondrial and endothelial mechanisms, whereas Prx6 exhibits dual roles shaped by its unique aiPLA2 activity and cellular origin. These findings underscore the therapeutic promise of targeting Prxs in a spatially and temporally nuanced manner to enhance endogenous protection while limiting inflammation.

## Figures and Tables

**Table 1:** Pharmacological modulation of Prxs in stroke.

Drug Name	Disease	Drug Mechanism	Target Prx Isoform	Drug Effect
Danhong [17]	ICH	Upregulation of Prx1	Prx1	Reduces brain edema, hematoma, and neuronal apoptosis; improves outcomes
Ligustilide [19]	ICH	Downregulation of Prx1	Prx1	Reduces neuronal apoptosis
Senkyunolide [19]	ICH	Downregulation of Prx1	Prx1	Reduces neuronal apoptosis
PJ34 (PARP-1 inhibitor) [42]	Ischemic stroke	Downregulation of Prx1	Prx1	Anti-inflammatory and improves outcomes
Prx1-targeted peptides [43]	Ischemic stroke	Downregulation of Prx1	Prx1	Reduces infarct size, neuronal apoptosis, and behavioral deficits
CD21 [50]	Ischemic stroke	Clearance of extracellular Prx1	Prx1	Improves outcomes
Gas-D [44]	Ischemic stroke	Inhibits TLR4/NF-κB	Prx1/2	Reduces inflammation and infarct size
Conoidin A [22]	ICH	Inhibition of Prx2	Prx2	Reduces tissue damage and improves outcomes
TAK242 (TLR4 inhibitor) [23]	ICH	Inhibition of TLR4	Prx2	Mitigates neuroinflammation and promotes recovery
Conoidin A [40]	IVH	Inhibition of Prx2	Prx2	Reduces inflammation, immune cell activation, and ventricular damage
Minocycline [40]	IVH	Anti-inflammatory	Prx2	Reduces inflammation, immune cell activation, and ventricular damage
CDk5 [53]	Ischemic stroke	Phosphorylates Prx2 at threonine	Prx2	Promotes neuronal death
Selenium [29]	ICH	Upregulation of Txnrd2/Trx2/Prx3 axis	Prx3	Reduces neuronal injury, brain edema, and improves outcomes
Recombinant Prx5 [37]	SAH	Supplementation	Prx5	Improves neurological outcomes, reduces brain edema, preserves BBB
Resveratrol [58]	Ischemic stroke	Maintains Prx5 expression	Prx5	Reduces oxidative damage and improves neuroprotection
4-Hydroxybenzoic acid [59]	Ischemic stroke	Activates PI3K/Akt signaling	Prx6	Enhances neuronal survival
4-Hydroxybenzyl alcohol [60]	Ischemic stroke	Upregulation of Prx6	Prx6	Provides neuroprotection
Curcumin [44]	Ischemic stroke	Upregulation of Prx6	Prx6	Reduces oxidative stress and infarct size
SCLC-conditioned medium [62]	Ischemic stroke	PTEN/PI3K/AKT signaling	Prx6	Alleviates apoptosis and oxidative damage
Melatonin [63]	Ischemic stroke	Antioxidant	Prx6	Neuroprotection
MJ33 [66]	Ischemic stroke	Inhibits the aiPLA2 enzymatic activity of Prx6	Prx6	Reduces infarct size and inflammation
Ligustilide [69]	Ischemic stroke	Inhibits TLR4, p-STAT3 and NF-κB signaling	Prx6	Reduces neurodegeneration

**Table 2:** Genetic approaches to studying Prxs in stroke.

Genetic Manipulation	Disease	Target Prx Isoform	Effect
Prx1-deficient mice [46]	Ischemic stroke	Prx1	Larger infarcts and worse outcomes
Prx2 knockdown [28]	ICH	Prx2	Exacerbates pyroptosis and neuronal injury
Prx2 knockdown [52]	OGD model	Prx2	Exacerbated neuronal injury
Prx2 T89A mutant (non-phosphorylatable) [53]	Ischemic stroke	Prx2	Neuroprotective
Phospho-mimic Prx2 mutant [53]	Ischemic stroke	Prx2	Not neuroprotective
Prx3 overexpression (via AAV) [32]	SAH	Prx3	Reduces oxidative stress, apoptosis, and enhances neuronal survival
Prx3 knockdown (via Txnrd2 siRNA) [29]	ICH	Prx3	Aggravates neurological deficits, brain edema, and neurological deficits
Prx4 knockdown [56]	Ischemic stroke	Prx4	Increases oxidative stress and inflammatory responses
Prx5 knockdown (siRNA) [37]	SAH	Prx5	Increases oxidative stress, apoptosis, and functional deficits
Prx6 knockdown [61]	Ischemic stroke	Prx6	Increase oxidative stress and infarct size
Prx6 knockdown [63]	Ischemic stroke	Prx6	Increases infarct volume, neuronal apoptosis, and mitophagy
Prx6 + PINK1 co-knockdown [63]	Ischemic stroke	Prx6	Decreases infarct volume, neuronal apoptosis, and mitophagy

## Data Availability

All supporting data are contained within the article.

## Abbreviations

The following abbreviations are used in this manuscript:

aiPLA2	Acidic, Ca^2+^-independent phospholipase A2
BBB	Blood–brain barrier
CDK5	Cyclin-dependent kinase 5
ChP	Choroid plexus
CRISPR	Clustered regularly interspaced short palindromic repeat
CSF	Cerebrospinal fluid
COX-2	Cyclooxygenase-2
DAMPs	Damage-associated molecular patterns
DNA	Deoxyribonucleic acid
HO-1	Heme oxygenase-1
hs-CRP	High-sensitivity C-Reactive Protein
ICH	Intracerebral hemorrhage
iNOS	Inducible nitric oxide synthase
IL	Interleukin
IVH	Intraventricular hemorrhage
MACO	Middle cerebral artery occlusion
MSC^CCR2	CCR2-engineered mesenchymal stem cells
NO	Nitric oxide
NF-κB	Nuclear factor kappa-light-chain-enhancer of activated B cells
Nrf2	Nuclear factor erythroid 2-related factor 2
OGD	Oxygen-glucose deprivation
RAGE	Receptor for advanced glycation end products
PARP-1	Poly (ADP-ribose) polymerase 1
PI3K	Phosphatidylinositol 3-kinase
Prx/Prdx	peroxiredoxin
ROS	Reactive oxygen species
RNA	Ribonucleic acid
PINK1	PTEN- induced kinase 1
PROZ	Protein Z
SAH	Subarachnoid hemorrhage
SAM	Stroke-associated microglia
SCLC	Schwann-like cell
siRNA	Small interfering RNA
TET-1	Tet methylcytosine dioxygenase 1
TNF-α	Tumor necrosis factor alpha
TLR	Toll-like receptor
tPA	Tissue plasminogen activator
Trx	Thioredoxin
Txnrd2	Thioredoxin-reductase 2
WFNS	World Federation of Neurosurgical Societies
